# A Parallelized Nanofluidic Device for High-Throughput Optical DNA Mapping of Bacterial Plasmids

**DOI:** 10.3390/mi12101234

**Published:** 2021-10-11

**Authors:** Sriram KK, Yii-Lih Lin, Tsegaye Sewunet, Marie Wrande, Linus Sandegren, Christian G. Giske, Fredrik Westerlund

**Affiliations:** 1 Division of Chemical Biology, Department of Biology and Biological Engineering, Chalmers University of Technology, 412 96 Gothenburg, Sweden; sriramk@chalmers.se (S.K.); yiilih@gmail.com (Y.-L.L.); 2 Division of Clinical Microbiology, Department of Laboratory Medicine, Karolinska Institute, 141 52 Stockholm, Sweden; tsegaye.sewunet@ki.se (T.S.); christian.giske@ki.se (C.G.G.); 3 Department of Medical Biochemistry and Microbiology, Uppsala University, 752 37 Uppsala, Sweden; marie.wrande@imbim.uu.se (M.W.); linus.sandegren@imbim.uu.se (L.S.); 4 Clinical Microbiology, Karolinska University Hospital, 171 76 Stockholm, Sweden

**Keywords:** nanofluidics, multiplexing, optical DNA mapping, plasmids, antibiotic resistance

## Abstract

Optical DNA mapping (ODM) has developed into an important technique for DNA analysis, where single DNA molecules are sequence-specifically labeled and stretched, for example, in nanofluidic channels. We have developed an ODM assay to analyze bacterial plasmids—circular extrachromosomal DNA that often carry genes that make bacteria resistant to antibiotics. As for most techniques, the next important step is to increase throughput and automation. In this work, we designed and fabricated a nanofluidic device that, together with a simple automation routine, allows parallel analysis of up to 10 samples at the same time. Using plasmids encoding extended-spectrum beta-lactamases (ESBL), isolated from *Escherichia* *coli* and *Klebsiella* *pneumoniae*, we demonstrate the multiplexing capabilities of the device when it comes to both many samples in parallel and different resistance genes. As a final example, we combined the device with a novel protocol for rapid cultivation and extraction of plasmids from fecal samples collected from patients. This combined protocol will make it possible to analyze many patient samples in one device already on the day the sample is collected, which is an important step forward for the ODM analysis of plasmids in clinical diagnostics.

## 1. Introduction

Optical DNA mapping (ODM) using fluorescence imaging of single long DNA molecules, pioneered by David Schwartz and others in the early 1990s [1,2], has now developed into a versatile tool to study genomic DNA. Early work involving DNA mapping was performed by stretching fluorescently labelled single DNA molecules on a positively charged or hydrophobic glass surface [3,4,5]. Although simple to use, the surface stretching of DNA molecules had drawbacks, in particular with non-uniform DNA stretching. The advent of micro- and nanofluidic devices opened a new possibility of stretching DNA molecules in highly confined environments, providing the opportunity to manipulate single DNA molecules that are not deposited on a surface [6,7,8,9].

Early studies of DNA molecules in nanochannels focused primarily on understanding the physics of confined DNA [9,10,11]. Soon after, research focusing on targeting the underlying DNA sequence was demonstrated, for example the interaction of the LacI repressor with specific sites on λ-DNA [12]. This, in turn, opened the possibility to use nanofluidic channels for ODM studies of DNA [13,14]. Various enzymatic labeling schemes, such as nick labeling [15], nick-flap labeling [16], and methyltransferase labeling [17], have been established and used successfully for ODM in nanochannels. We have developed an enzyme-free ODM assay, based on one-step competitive binding that creates an emission intensity variation along the DNA molecule [14,18]. For this, the AT-specific drug netropsin and the fluorescent dye YOYO-1 are added to the DNA at the same time, with netropsin:YOYO-1 ratios around 100:1. Netropsin binds to AT-rich regions, leaving the non-specific YOYO-1 to bind primarily to the GC-rich regions, thus creating an intensity variation along the DNA, a “DNA barcode” [14]. This labeling protocol has proved to be useful for various applications, such as plasmid characterization [19,20] and typing of bacteria [21], as well as human genome analysis [22].

According to the World Health Organization (WHO), antimicrobial resistance (AMR) is one of the top ten global threats to human health. AMR imposes a huge present and future burden on global health and economy and requires immediate attention to the development of new diagnostic tools and antibiotics [23]. One key area where competitive binding-based ODM has been successfully used is the study of plasmids, circular DNA molecules not part of the bacterial chromosome, that play an important role in the spread of AMR. By combining the ODM technique with Cas9-mediated identification of resistance genes, the method can determine the number of different plasmids in a sample, their size, on which plasmid a certain resistance gene is located and also use the ODM barcode to identify and trace plasmids [20]. Our methodology has proven to be useful in understanding the role of plasmid conjugation, for example, in hospital outbreaks and recurring urinary tract infections [19,24,25,26,27].

To use the assay in a clinical microbiology lab, it is necessary to investigate hundreds of samples per day. In our previous studies, we used a setup consisting of a single set of nanochannels where the experiments for one sample can be completed in approximately one hour. This means that, on a typical workday, up to eight samples could be analyzed.

In this study, we developed a nanofluidic device with the capability to perform ODM on ten different samples at the same time and, with the help of a simple pressure pump and epi-fluorescence microscope, we were able to automate the image acquisition process, thus minimizing the time spent by the researcher in collecting the data. We demonstrate the principle of the device using model DNA. We then turn to the analysis of bacterial plasmids containing important resistance genes from different bacterial species. Finally, we combine the assay with a rapid protocol for extracting plasmids from fecal samples, which is an important step towards clinical use of the technique.

## 2. Materials and Methods

### 2.1. Nanofluidics and System Automation

**Figure 1:**
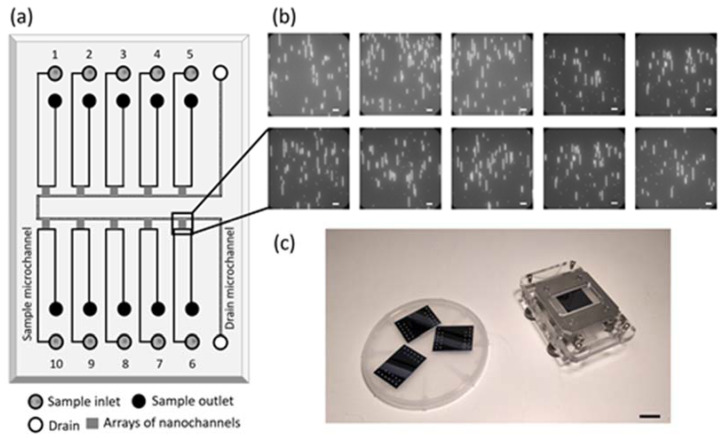
(**a**) Schematics of the multiplexed nanofluidic chip with inlet, outlet and drain. (**b**) Fluorescence images of single DNA molecules obtained simultaneously from the ten different nanochannel modules of the chip. Scale bar: 15 µm. (**c**) Image showing the chips and the chip holder enabling automation of pressure-driven flow and ODM using an epi-fluorescence microscope. Scale bar: 20 mm.

The nanochannels are of the dimensions of 100 × 150 nm^2^ (width × height) and there are 400 nanochannels in each set (Figure 1). Each nanochannel set is connected to their own inlet microchannel and a shared outlet microchannel (drain). The nanochannels were fabricated with e-beam lithography, followed by the fabrication of microchannels with standard photolithography. Sample loading holes were etched via a deep-RIE process and wafer scale fusion bonding was performed to obtain the micro- and nanoconfinements; the wafer was finally diced to obtain the individual chips [10,28]. The chips were then mounted on an acrylic chuck, allowing pre-loading of the sample solutions. This entire setup was custom-designed to fit on an epi-fluorescence microscope (Zeiss AxioObserver.Z1, Carl Zeiss AG, Jena, Germany) and has valves to apply N_2_-pressure with the help of a tabletop pressure pump (Elveflow OB1 MK3, Paris, France). The setup was automated using a simple program written within the Elveflow software, which was then designed to trigger the EMCCD camera (Andor Ixon 888, Oxford Instruments, Abingdon, UK) to collect images. The Zeiss imaging software (Zen pro blue edition) was programmed to collect images (20 frames, 100 ms per frame) by moving the microscope stage to designated locations, thereby collecting one image from each of the ten nanochannel modules. Then, the pump flushes the DNA molecules out and brings new molecules in for the next set of images to be taken. This process is repeated until a sufficient amount of data is collected.

### 2.2. Sample Preparation

#### 2.2.1. λ-DNA at Different Ionic Strengths

For the experiments at different ionic strengths, 5 µM λ-DNA (48502 bp, New England Biolabs, Ipswich, MA, USA) was mixed with 2 µM YOYO (Invitrogen, Waltham, MA, USA) and 520 µM netropsin (Sigma-Aldrich, St. Louis, MO, USA) in Tris-Borate-EDTA buffer (TBE, Sigma-Aldrich, St. Louis, MO, USA); the samples were incubated at 50 °C for 30 min and diluted in MilliQ water to obtain the different ionic strengths. 3% (*v*/*v*) β-mercaptoethanol (Sigma-Aldrich, St. Louis, MO, USA) was added to suppress photonicking and photobleaching.

#### 2.2.2. Plasmid Samples for ODM

Bacterial strains were cultured for 12–16 h in LB broth supplemented with ampicillin (30 µg/mL) and plasmids were extracted using a NucleoBond Xtra Midi Kit (Macherey-Nagel GmbH, Düren, Germany), according to the manufacturer’s instructions. The eluted DNA was precipitated with 70% isopropanol and reconstituted with TE buffer (Sigma-Aldrich, St. Louis, MO, USA). The plasmids were treated by either Cas9 (PNA Bio, Thousand Oaks, CA, USA) with tracrRNA (Dharmacon Inc., Lafayette, CO, USA) and crRNA (Dharmacon Inc., Lafayette, CO, USA), targeting specific antibiotic resistance genes (*bla*_CTX-M-14_ or *bla*_CTX-M-15_), or just the tracrRNA, without crRNA as control, as shown in the proof-of-concept experiments in Section 3.4. The sequences of the RNA targeting the *bla*_CTX-M-14_ and *bla*_CTX-M-15_ genes were 5′AGAGAGCCGCCGCGATGTGC3′ and 5′CCGTCGCGATGTATTAGCGT3′, respectively. After the Cas9 reaction, the plasmids were mixed with λ-DNA as internal size reference and then fluorescently stained with YOYO-1 and netropsin. The molar ratio of YOYO-1 to DNA was 1:2, while that of netropsin to YOYO-1 was 70:1; the staining was performed in 0.5x TBE buffer for 30 min at 50 °C. The samples were then diluted to 0.05x TBE to optimize the DNA stretching in the nanochannels [13]. Β-mercaptoethanol was added at 3% (*v*/*v*) to suppress photonicking and photobleaching.

#### 2.2.3. Clinical Fecal Samples

Five fecal samples (F1–F5) known to contain extended-spectrum beta-lactamase-producing Enterobacterales were collected. The samples were inoculated in 100 mL of LB broth supplemented with ampicillin (30 µg/mL) and incubated at 37 °C in a shaking incubator (300 rpm) for 4 h. Plasmid extraction was performed using the NucleoBond Xtra Midi (Macherey Nagel GmbH, Düren, Germany) plasmid extraction and purification kit, as in Section 2.2.2.

### 2.3. Data Analysis

The images collected were first converted into multi-TIFF files. The data obtained from each nanochannel module were separated, to process data from the different samples independently. The image data were then processed to identify fluorescent DNA molecules and their corresponding parameters, including size and the sequence-specific barcodes, as well as the potential presence of resistance genes (*bla*_CTX-M-14_ or *bla*_CTX-M-15_). More details on image analysis for ODM has been discussed in our earlier studies [18,20,29]. Briefly, the identified DNA molecules were first sub-grouped by their mean intensity and sizes, compared with λ-DNA as a reference. Sub-groups of DNA molecules that are twice brighter than λ-DNA are circular plasmids [30]. Sub-groups of linearized plasmids typically have similar intensity as λ-DNA and their sizes are 1.8 times longer than their circular counterparts [13,30]. Sub-grouped linearized DNA molecules with similar sizes and intensities were then compared for barcode similarity using Pearson cross correlation and merged to a consensus barcode. The barcodes were further used for comparison among isolates (using a *p*-value approach [27,29]) and for confirming the presence of resistance genes by identifying consensus double-stranded breaks by Cas9.

For the comparison of experimental barcodes with long-read sequencing in Section 3.4, FASTA files were converted into a “barcode” based on the binding affinity of YOYO-1 and netropsin to the underlying sequence. The entire procedure has been discussed in detail in our earlier work [18].

## 3. Results and Discussion

### 3.1. Nanofluidic Device

In this work, we used standard semiconductor fabrication methods to make our devices [10]. To start with, computer-aided designing (AutoCAD 2020, Autodesk Inc. San Rafael, CA, USA) was used to prepare the design layout. Nanochannels, microchannels and sample loading holes were designed and the dimensions were chosen to fit the acrylic sample holder on which the fluidic device was mounted, enabling N_2_ pressure-driven flow of DNA molecules from the sample loading holes to the nanochannels and fluorescence imaging of the stretched DNA molecules in the nanochannels.

Each device consists of ten microfluidic channels, each having an inlet and an outlet to assist sample loading and manipulation of DNA molecules in the microchannels (Figure 1). A common drain-microchannel connects the other end of all the ten sets of nanochannels. The nanochannel sets were designed with the travelling distance of the microscope objective in mind and to minimize out-of-focus issues when the objective automatically moves from one nanochannel set to the next. A total of 400 parallel nanochannels allows tens of DNA molecules to be stretched at the same time and each nanochannel set spans over an area of 300 × 500 µm^2^, which makes it possible to image DNA molecules in different regions of the same nanochannel set.

### 3.2. Automation of Pressure-Driven Flow and Imaging

The entropic barrier between the microchannels and the nanochannels allows the DNA to be preconcentrated in front of the nanochannels at a low applied pressure (400 mbar) in order to maximize the number of DNA molecules per field of view (see Materials and Methods). After preconcentration, the DNA molecules were pushed into nanochannels with a short pulse of high pressure (2000 mBar, 800 ms), sufficient to overcome the entropic barrier and short enough to keep the DNA molecules within the 500 µm long nanochannels. The pressure cycles to flush DNA through the microchannels, preconcentrate at the entrance of the nanochannels and push into the nanochannels were repeated and followed by automated imaging (see Materials and Methods).

**Figure 2:**
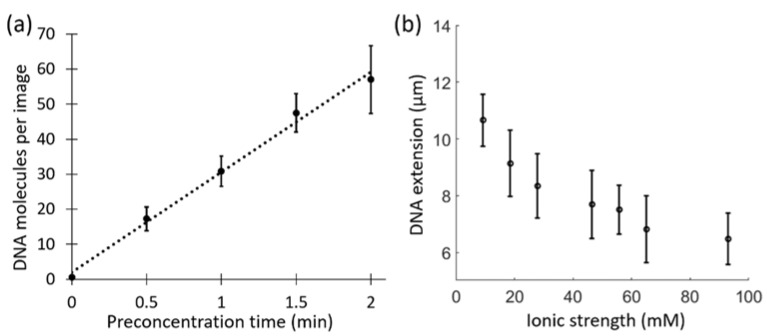
(**a**) The number of λ-DNA molecules collected per image with increasing preconcentration time. Error bars are the standard deviations from five images per time point. (**b**) λ-DNA extension at seven different ionic strengths measured simultaneously. Each point consists of data for at least 100 molecules. Error bars are standard deviations.

To start with, we performed an experiment to evaluate the effect of preconcentration time on the number of DNA molecules obtained per image in our setup. We used preconcentration times from 30 s to 2 min and found that the number of λ-DNA molecules per image increased linearly with time. For 2 min of preconcentration, we were able to image ~60 λ-DNA molecules per image (Figure 2a). Increasing the preconcentration time further led to that the channels were too crowded with DNA to allow single DNA molecules to be imaged. It is worth noting that the starting DNA concentration and the image field of view also contributes to the number of DNA molecules obtained per image. For all further experiments, a preconcentration time of 2 min was used.

### 3.3. DNA Stretching at Varying Ionic Strengths

With an optimized automation procedure for DNA accumulation and image acquisition, next, we performed a proof-of-concept experiment with λ-DNA molecules at different ionic strengths. λ-DNA in TBE buffer at varying ionic strengths (between 10 mM and 100 mM) was loaded into the different nanochannel modules of the device. The independent nanochannel modules made it possible to study all the different ionic strength conditions at the same time in a single experiment. Images were collected for each ionic strength and the extensions of the DNA molecules were determined from at least 100 λ-DNA molecules at each ionic strength. We found that the DNA stretching, as expected, decreased with the increase in ionic strength (Figure 2b [31,32,33]).

### 3.4. Proof-of-Concept ODM Experiment

**Figure 3:**
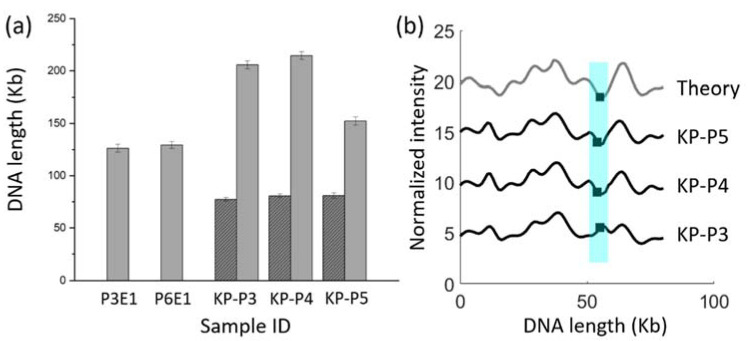
(**a**) Lengths of plasmids found in the five different isolates. Dark gray-shaded bars represent plasmids where the *bla*_CTX-M-15_ gene was identified. (**b**) Barcodes of ~80 kb plasmids with the *bla*_CTX-M-15_ gene in the KP-P3, KP-P4 and KP-P5 isolates (black), compared to the theoretical barcode generated from long-read sequencing (gray). Black squares within the highlighted region represent the position of the *bla*_CTX-M-15_ gene.

Next, we turned to demonstrating ODM of plasmids carrying the antibiotic resistance gene *bla*_CTX-M-15_. We selected isolates that were part of a previously published study, where the aim was to characterize plasmids during an outbreak of extended-spectrum β-lactamase (ESBL)-producing *Enterobacteriaceae* at a Swedish neonatal intensive care unit [19]. From this set of isolates, we picked two ESBL-producing *E. coli* (ESBL-EC) isolates (P3E1 and P6E1) that did not carry the *bla*_CTX-M-15_ gene of interest on a plasmid and three ESBL-producing *K. pneumoniae* (ESBL-KP) isolates (KP-P3, -P4 and -P5) with the *bla*_CTX-M-15_ gene on a plasmid. For all five isolates, two samples were loaded in separate nanochannel modules in the multiplexed device, one treated with Cas9 targeting the *bla*_CTX-M-15_ gene and one without. P3E1 and P6E1 had plasmids of identical sizes (~130 kb). KP-P3, KP-P4 and KP-P5 each had two plasmids, one of identical size in all isolates (~80 kb, represented by the dark gray-shaded bars in Figure 3a) and one much larger that varied in size among the isolates (represented by the light gray bars in Figure 3a). After the Cas9 restriction, we found that the isolates P3E1 and P6E1 showed no plasmids linearized by Cas9, in line with the fact that the plasmids found in these isolates did not carry the *bla*_CTX-M-15_ gene [19]. However, for the isolates KP-P3, KP-P4 and KP-P5, we identified the *bla*_CTX-M-15_ gene on the ~80 kb plasmid, in accordance with our previously reported results [19]. A comparison of the barcode between the plasmids in these three isolates also showed that they were identical and that the location of the *bla*_CTX-M-15_ gene agreed with long-read sequencing (Figure 3b).

### 3.5. Identification of Multiple Resistance Genes on Plasmids

**Figure 4:**
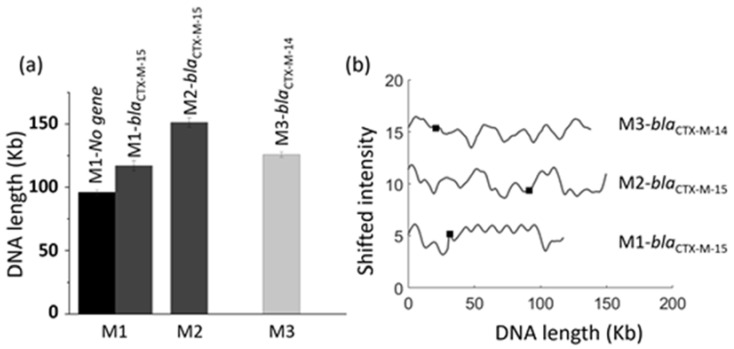
(**a**) Sizes of plasmids and expected resistance genes found in isolates M1, M2 and M3. (**b**) Barcodes of plasmids with the *bla*_CTX-M-14_ or *bla*_CTX-M-15_ gene in these isolates. Black squares represent the position of the gene. The barcodes are shifted vertically for clarity.

To further demonstrate the usefulness of the multiplexed device, we extended our study to detect different resistance genes at the same time. For this, we used isolates M1, M2 and M3, where we expected to have plasmids carrying either of the two resistance genes *bla*_CTX-M-14_ or *bla*_CTX-M-15_ [34]. Each isolate was prepared twice, one with gRNA targeting *bla*_CTX-M-14_ and the other targeting *bla*_CTX-M-15_ (Figure 4). The sizes of all the plasmids identified in these three isolates are shown in Figure 4a and the barcodes of the plasmids carrying either the *bla*_CTX-M-14_ or *bla*_CTX-M-15_ gene are presented in Figure 4b. All three isolates had plasmids of different sizes and barcodes and we identified no similarity between them.

### 3.6. Antibiotic Resistance Gene Identification in Clinical Fecal Isolates

Detection of antibiotic resistance genes located on plasmids in bacterial isolates from clinical specimens can take more than two days if the bacterial colonies are to be first identified and targeted for plasmid extraction. Alternatively, the sample can be directly inoculated into the appropriate medium supplemented with antibiotics suitable for selection of the resistant isolates. Then, after overnight incubation, plasmids can be extracted and purified. However, this approach increases the turnaround time when using conventional methods. Herein, we used a modified enrichment protocol which is four-times faster than the standard enrichment protocol (see Materials and Methods), resulting in the workflow of the whole analysis being reduced to eight hours. The principle of this brief enrichment is to remove irrelevant bacterial species and enrich bacteria carrying the AMR plasmids of interest, thus enabling plasmid extraction from liquid culture instead of bacterial colonies.

**Figure 5:**
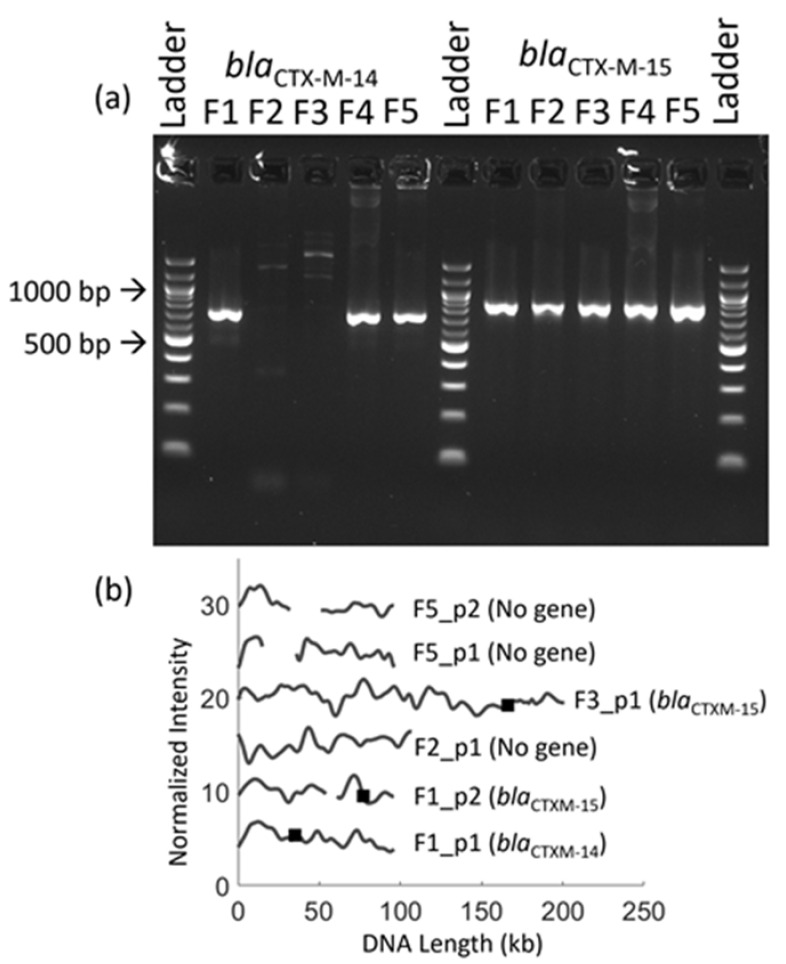
Identification of *bla*_CTX-M-14_ and *bla*_CTX-M-15_ genes in clinical fecal samples. (**a**) Results from PCR for clinical fecal isolates F1–F5 for genes *bla*_CTX-M-14_ and *bla*_CTX-M-15_. (**b**) Barcodes for clinical fecal samples F1, F2, F3 and F5 showing the presence or absence of the targeted resistance genes on plasmids, the gene identified and its location (black square) along the DNA barcode. The barcodes are shifted vertically for clarity.

We selected five clinical fecal samples (F1–F5) and extracted plasmids using the four-hour enrichment protocol. We performed a polymerase chain reaction (PCR) followed by agarose gel electrophoresis that revealed that F1, F4 and F5 encoded the *bla*_CTX-M-14_ gene and that all five samples encoded the *bla*_CTX-M-15_ gene (Figure 5a). The quality of sample F4 was not good enough to proceed with the ODM experiments. We investigated the remaining four samples using ODM, each sample being studied for the *bla*_CTX-M-14_ and *bla*_CTX-M-15_ genes (Figure 5). In sample F1, two plasmids were found with *bla*_CTX-M-14_ on a ~96 kb plasmid and *bla*_CTX-M-15_ on a ~85 kb plasmid, in accordance with the PCR results. The ODM analysis confirmed that the two plasmids were different. Sample F2 had only one plasmid and sample F5 had two plasmids, but ODM suggested that neither of the two resistance genes were located on these plasmids. This could be due to the resistance genes being present in the chromosomal DNA instead. Isolate F3 had a large plasmid (~200 kb) carrying the *bla*_CTX-M-15_ gene, in accordance with the PCR results. An important difference between PCR and ODM is that, while the PCR analysis reveals the presence of a specific gene, the ODM analysis determines on which plasmid a specific gene is located. This is important, for example, for epidemiological studies of bacterial transmission [19] and plasmid conjugation [24,26]; we have also shown, previously, that detecting identical plasmids in several samples can be a proxy for ongoing clonal outbreaks [25,27].

## 4. Conclusions

In this work, we demonstrate the design and fabrication of a nanofluidic chip with ten separately addressable modules, where the device can be filled and images acquired automatically. The device can be used for fundamental studies of the physical properties of DNA, as demonstrated here by simultaneously studying λ-DNA molecules in buffers of various ionic strengths. More importantly, the device is perfectly suited for simultaneous analysis of many clinical samples, illustrated here by analysis of bacterial plasmids carrying antibiotic resistance genes. The multiplexing can be performed either to study samples from different patients simultaneously, or to analyze the presence of different resistance genes at the same time, or a combination thereof. With our traditional devices with one nanochannel module, one sample can be analyzed per hour. With our new multiplexed and automated chip, ten samples can be analyzed per hour, which drastically reduces the time per sample, and at least a hundred samples could be analyzed per device per day. In a final example, we demonstrate a possible clinical application where we decreased the time for the enrichment of bacteria from fecal samples, which means that the analysis can be completed on the same day the sample is collected. This is a crucial improvement for future clinical use of the assay for plasmid analysis, but could also be used for, for example, bacterial identification [21].

## Data Availability

All data and information related to device design/schematics can be obtained from the corresponding author upon reasonable request.
